# The Effect of Low Glycemic Load Diet on Central Obesity and Lipid Related Metabolic Abnormalities in T2DM Patients Is Comparable to That of Energy‐Restricted Diet: A Randomized, Open, Parallel Controlled Study

**DOI:** 10.1002/fsn3.71995

**Published:** 2026-06-09

**Authors:** Xiuhong Lin, Chaogang Chen, Meng Ren, Chulin Huang, Diaozhu Lin, Cheng Wang, Xiaoting Lu, Xiaoyi Wang, Dan Liu, Jin Zhang, Li Yan, Mingtong Xu

**Affiliations:** ^1^ Department of Clinical Nutrition Sun Yat‐Sen Memorial Hospital, Sun Yat‐Sen University Guangzhou Guangdong People's Republic of China; ^2^ Department of Endocrinology Sun Yat‐Sen Memorial Hospital, Sun Yat‐Sen University Guangzhou Guangdong People's Republic of China

**Keywords:** atherosclerosis index, glycemic load, intensive insulin therapy, Type 2 diabetes, visceral adiposity index, weight‐corrected waist index

## Abstract

Type 2 diabetes mellitus (T2DM) is closely associated with central obesity, which further elevates cardiovascular disease risk. This randomized, open‐label, parallel‐controlled trial aimed to compare the effects of a low glycemic load (LGL) diet and a conventional energy‐restricted diet on central obesity and lipid‐related metabolic abnormalities in newly diagnosed T2DM patients following short‐term intensive insulin therapy (SIIT). All participants received a 2‐week course of SIIT before being randomly allocated to either the control group receiving a conventional energy‐restricted diet (25–30 kcal/kg/day, with 45%–55% of energy from carbohydrates, 15%–20% from protein, and 25%–35% from fat) or an energy‐matched LGL group with a target glycemic load of 56–68/1000 kcal. The 1‐year dietary intervention was implemented with quarterly outcome assessments, and repeated‐measures data were analyzed using generalized estimating equations via R 4.3.2. Between 2019 and 2023, a total of 366 patients were screened, and finally 89 eligible participants were enrolled, including 45 cases in the control group and 44 in the LGL group. During follow‐up, significant intergroup differences were observed in dietary composition (GL/1000 kcal and macronutrient distribution) and changes in HbA1c. Energy intake, physical activity level (MET‐h/week), body mass index, waist circumference, weight‐corrected waist circumference, visceral adiposity index, and atherosclerosis index were all markedly reduced from baseline in both groups (*p*_time_ < 0.05), while no significant between‐group differences were detected for these indicators (*p*_group_ > 0.05). In conclusion, the LGL diet and conventional energy‐restricted diet exert comparable effects in improving central obesity and metabolic profiles among newly diagnosed T2DM patients after SIIT.

**Trial Registration:** ChiCTR1900020559

## Graphical abstract

This graphical abstract illustrates the design and key findings of a randomized, open‐label, parallel‐controlled trial comparing two dietary maintenance strategies in newly diagnosed Type 2 diabetes mellitus (T2DM) patients after short‐term intensive insulin therapy (SIIT). (A) Study flow: between 2019 and 2023, 366 newly diagnosed T2DM patients were screened, and 89 eligible patients were randomly assigned after SIIT: 45 to the conventional energy‐restricted control diet group and 44 to the low glycemic load (LGL) diet group. Both groups received 1 year of dietary intervention with quarterly evaluations. (B) Dietary Interventions: The control diet provided 25–30 kcal/kg/day with standard macronutrient distribution (45%–55% carbohydrate, 15%–20% protein, 25%–35% fat). The LGL diet was energy‐matched but designed to control glycemic load within 56–68/1000 kcal, using low‐GI food choices. (C). HbA1c changes over time: both groups showed significant reductions in HbA1c over the 12‐month follow‐up period (*p*_time_ < 0.05), with the LGL group demonstrating a significantly greater reduction compared to the control group (*p*_group_ < 0.05). (D) Central obesity and lipid markers improvement: Both dietary regimens significantly improved central obesity (WWI) and lipid‐related markers (VAI, AI) over time (*p*_time_ < 0.05), with no significant differences observed between the two groups (*p*_group_ > 0.05). These findings highlight that both conventional energy‐restricted and LGL diets are effective maintenance strategies for post‐SIIT T2DM patients. While energy restriction remains the core driver of metabolic improvement, the LGL diet offers an additional benefit in glycemic control, providing a flexible, personalized nutritional option for clinical practice.

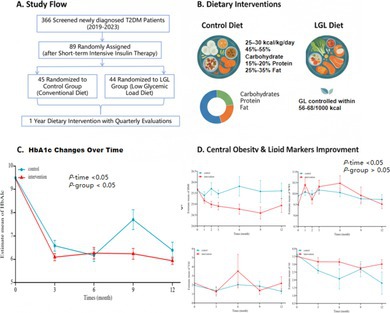

**Abbreviations**
ADA: American Diabetes Association
AI: atherosclerosis index
ALT: glutamic‐pyruvic transaminase
AUC: area under the curve
BMI: body mass index
CHO: carbohydrate
CHO%: carbohydrate percentage of energy supply
CSII: continuous subcutaneous insulin infusion
DBP: diastolic blood pressure
FAS: full analysis set
FAT%: fat percentage of energy supply
FBG: fasting blood glucose
FINS: fasting plasma glucose
FPG: fasting plasma glucose
GA: glycated albumin
GI: glycemic index
GL: glycemic load
HbA1c: glycosylated hemoglobin A1c
HDL‐C: high‐density lipoprotein cholesterol
HOMA‐IR: homeostasis model evaluation of insulin resistance
HOMA‐β: homeostasis model evaluation of basal insulin secretion
Ht: height
INS: insulin
IR: insulin resistance
ITT: intent‐to‐treat analysis
LDL‐C: low‐density lipoprotein cholesterol
LGL: low glycemic load
MET‐h: metabolic equivalent‐hours of exercise
OR: odds ratio
PBG: postprandial blood glucose
PG: plasma glucose
PPS: per protocol set
PRO: protein
PRO%: protein percentage of energy supply
RCT: randomized controlled trial
SBP: systolic blood pressure
Scr: serum creatinine
SIIT: short‐term insulin intensive therapy
T2DM: Type 2 diabetes mellitus
TBA: total bile acid
TC: total cholesterol
TG: triglyceride
TIR: time in range (PG 3.9–10.0 mmol/L)
TSH: thyroid‐stimulating hormone
VAI: visceral adiposity index
Wc: waist circumference
Wt: weight
WWI: weight‐adjusted waist circumference index

## 1 Introduction

Diabetes is a global chronic disease with a continuously rising prevalence rate, especially in China (Zhang, Wu, et al. 2024). It is often accompanied by central obesity and lipid metabolism disorders, which increase the risk of cardiovascular complications (Mengstie et al. 2024; Hara et al. 2024). Central obesity, especially an enlarged waist circumference, is regarded as a key pathophysiological feature and is closely related to deteriorated blood glucose control, insulin resistance (IR), and dyslipidemia (Zhou et al. 2025; Song et al. 2023). Epidemiological studies have shown that about 60%–80% of Type 2 diabetes mellitus (T2DM) patients have central obesity (Li et al. 2023; Wang et al. 2024). Although traditional measures such as body mass index (BMI) and waist circumference (Wc) are widely used to assess obesity, they are insufficiently sensitive to ectopic deposition of visceral fat and are susceptible to muscle mass and body size (Sun et al. 2023). In recent years, weight‐corrected waist index (WWI), as a new central obesity index, has gradually attracted attention because it reduces the bias of individual body shape differences by correcting weight. Studies have shown that WWI is significantly positively associated with the risk of T2DM, and its discriminant ability is better than BMI and Wc (higher AUC values) when predicting the onset of diabetes (Wu et al. 2025; Wu and Guo 2024). In addition, longitudinal cohort analysis suggests that every unit increase in WWI increases the risk of T2DM by about 30%, which is more predictive especially in Asian populations (Li et al. 2024). In prospective cohort studies involving patients with Type 2 diabetes, WWI was positively correlated with all‐cause mortality and cardiovascular mortality. Based on findings from European and American populations, for every 1‐unit increase in WWI, the all‐cause mortality rate of T2DM patients significantly rose (HR 1.12–1.14) (Zhang, Zhang, et al. 2024).

In assessing visceral fat function and metabolic risk, visceral adiposity index (VAI) more accurately reflects visceral fat accumulation and its metabolic toxicity by integrating parameters such as waist circumference, BMI, triglycerides, and high‐density lipoprotein cholesterol (HDL‐C) (Zakerkish et al. 2023; Feng et al. 2024). Meta‐analysis confirmed that there is a dose–response relationship between VAI and T2DM risk, with a 12%–18% increase in T2DM risk for every 1 unit rise (Shen et al. 2024; Zhou et al. 2024). In addition, VAI is outstanding in predicting diabetic complications, as it is independently associated with cardiovascular events and diabetic nephropathy risk, and its discriminant ability is superior to traditional abdominal obesity indicators (such as WC, WHR) (Qiao et al. 2022; Zhou et al. 2023). Moreover, atherosclerosis index (AI), the ratio of triglycerides to HDL‐C, which serves as an indicator of lipid metabolism disorders and cardiovascular risk, has important prognostic significance in patients with T2DM. AI is significantly associated with the risk of carotid plaque and coronary artery calcification in patients with T2DM (Wang et al. 2025). Studies have shown that patients with elevated AI have a 1.5–2‐fold increase in the risk of atherosclerotic cardiovascular disease (ASCVD) in 10 years, especially in T2DM patients with central obesity. AI is independently associated with cardiovascular mortality (Zheng et al. 2023). These evidences indicate that the combined application of WWI, VAI, and AI indicators can evaluate metabolic abnormalities and long‐term prognosis in patients with T2DM in multidimensional ways and provide a basis for individualized intervention (Tao et al. 2024; Duan et al. 2024; Yang et al. 2024).

In diabetes management, nutritional intervention is one of the core strategies. A low glycemic load (LGL) diet aims to stabilize blood glucose fluctuations and improve metabolic health by improving quality and reducing quantity of carbohydrates in the diet (Peres et al. 2023; Gerontiti et al. 2024; Manta et al. 2023; Ellison et al. 2024). Meanwhile, a conventional energy‐restricted balanced diet is often used for weight control and blood glucose management (Al Sifri et al. 2024). Existing evidence suggests that LGL diets may improve metabolic outcomes by reducing IR and regulating lipid parameters such as low‐density lipoprotein cholesterol (LDL‐C) and triglycerides (TGs) (Anyang Kaakyire et al. 2025; Tian et al. 2024; Li and Yuan 2022) while conventional energy‐restricted balanced diets have been shown to be beneficial for weight loss and blood glucose improvement under overall calorie restriction (Ellison et al. 2024; Al Sifri et al. 2024; Geurts et al. 2023). However, there is a lack of consistent evidence on the direct comparison of LGL diets and conventional energy‐restricted balanced diets in terms of their effects on central obesity and lipid metabolism‐related indicators in diabetic patients. Some studies have shown controversial or uncertain results (Wong et al. 2024; Ichikawa et al. 2024). For instance, a systematic review pointed out that the comparison between low‐carbohydrate diets (usually associated with LGL) and low‐fat diets (similar to conventional energy‐restricted balanced diets) may show different effects on lipid metabolism due to population heterogeneity (Ellison et al. 2024; Anyang Kaakyire et al. 2025). Additionally, long‐term intervention studies suggest that the effectiveness of LGL diets in blood glucose control is controversial, and their specific impact on central obesity has not been fully evaluated in direct comparisons (Hara et al. 2024; Ichikawa et al. 2024). Therefore, current research urgently needs to fill this knowledge gap to optimize individualized nutritional strategies for diabetic patients.

Against this background, this study aimed to compare the effects of a LGL diet versus a conventional energy‐restricted diet on central obesity and lipid‐related metabolic abnormalities in newly diagnosed T2DM patients after SIIT over a 1‐year intervention period, so as to provide clinical evidence for optimizing nutritional intervention strategies for this specific population.

## 2 Materials and Methods

### 2.1 Research Design

**FIGURE 1:**
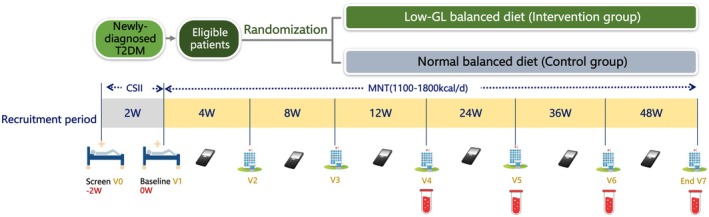
Flow chart of research design. This study was a randomized, open‐label, parallel controlled trial in newly diagnosed T2DM patients. It was conducted in two phases: patients who qualified for primary screening were hospitalized for CSII for about 2 weeks, and those who were re‐evaluated and met the enrollment criteria were randomly assigned to a low‐GL dietary intervention group and a control group for 1 year after discharge. The daily diet of patients was guided and supervised by the software. During the first 3 months of follow‐up, patients were visited once a month to assess their diet and blood glucose, and HbA1c and lipid profile were reviewed every 3 months. CSII: continuous subcutaneous insulin infusion, GL: glycemic load, MNT: medical nutrition therapy, T2DM: Type 2 diabetes mellitus.

This was a single‐center, randomized, open‐label, parallel‐controlled trial involving newly diagnosed T2DM patients at the Sun Yat‐sen Memorial Hospital of Sun Yat‐sen University starting January 2019. It should be noted that the current study is a sub‐study of a larger parent trial. The primary aim of the parent trial was to assess whether a LGL diet enhances diabetes remission rates after short‐term intensive insulin therapy (SIIT) in patients with newly diagnosed T2DM. The intervention of the parent trial, which was also adopted in this substudy, comprised SIIT for 2 weeks in the hospital followed by a 1‐year dietary intervention after discharge (Figure 1). The current substudy focuses on comparing the effects of the LGL diet versus the conventional energy‐restricted diet on central obesity and lipid‐related metabolic abnormalities in the same cohort of newly diagnosed T2DM patients after SIIT, which is a secondary objective derived from the parent trial.

### 2.2 Subjects

#### 2.2.1 Inclusion Criteria

Participants aged 18–65 years, newly diagnosed with T2DM according to ADA 2010 diagnostic criteria, negative for pancreatic islet autoantibodies, never taken hypoglycemic drugs, and underwent SIIT for 7–10 days after achieving target blood glucose levels. Participants were required to adhere to regular follow‐ups.

#### 2.2.2 Exclusion Criteria

Patients with Type 1 diabetes, severe diabetic complications (including diabetic ketoacidosis, lactic acid poisoning events, hypertonic coma, intermittent claudication, or diabetic foot) or comorbidities (such as active liver disease, renal insufficiency, cardiac dysfunction, stroke, or malignant tumor), history of depression or drug abuse, pregnant or lactating women.

#### 2.2.3 Exit Criteria

Subjects were withdrawn if they experienced elevated fingertip blood glucose (more than 50% of fasting blood glucose (FBG) ≥ 7.0 mmol/L or 2‐h postprandial blood glucose (PBG) ≥ 10.0 mmol/L) persisting over 1 week or HbA1c ≥ 7.0% during the follow‐up period. Participants were also excluded if lost to follow‐up due to illness or personal reasons.

### 2.3 Sample Size

This study is a subanalysis of the above‐registered RCT, and the sample size is derived from the design plan of the main study. The main study took the remission rate of diabetes as the primary efficacy indicator and adopted a 1:1 equal superiority design. The required sample size was calculated using the following statistical formula, where *P*_1_ is the outcome event rate of the first group, *P*_2_ is the outcome event rate of the second group, the significance level *α* is two‐sided 0.05, and the test level was set with a power of 1 − *β* = 0.8. Considering a 20% dropout rate, the required sample size was determined to be 86 cases, with 43 cases in each group.

$n={\frac{\left[Z_{\alpha }\sqrt{2\bar{p}\left(1-\bar{p}\right)}+Z_{\beta }\sqrt{P_{1}\left(1-P_{1}\right)+P_{2}\left(1-P_{2}\right)}\right]}{{\left(P_{1}-P_{2}\right)}^{2}}}^{2}$

### 2.4 Randomization

Block randomization was used for assigning participants to groups. Random numbers were generated using the SAS 9.4 software, block length set at 4. Two higher numbers designated participants to the control group, two lower numbers to the intervention group. Assignments were sealed in opaque envelopes for secure storage.

### 2.5 Intervention Program

#### 2.5.1 First Stage (−2 to 0 Weeks): SIIT

All participants were hospitalized for about 2 weeks to receive continuous subcutaneous insulin infusion (CSII) using Insulin Glulisine (Sanofi, France) administered through an insulin pump (Minimed712, Medtronic, USA). Starting insulin dose ranged from 0.4 to 0.6 IU/kg/day, evenly divided between basal and premeal doses. Insulin adjustments were made aiming to achieve target blood glucose levels (FBG < 6.1 mmol/L or 2‐h PBG < 7.8 mmol/L) within first 3 days, then maintaining CSII for 7–14 days. Hypoglycemia, defined as blood glucose below 3.9 mmol/L, and severe hypoglycemia, below 2.8 mmol/L, necessitated interventions such as carbohydrate ingestion, glucagon, or other resuscitation treatments.

All subjects were educated on controlling daily target energy intake (25–30 kcal/kg of ideal body weight) and nutrient distribution (with an energy supply ratio of 45%–60% carbohydrates, 15%–20% protein, and 20%–35% fat) according to the *2024 Chinese Diabetes Medical Nutrition Treatment Guidelines* (Diabetes Society of the Chinese Medical Association 2025).

#### 2.5.2 Second Stage (0–48 Weeks): Dietary Intervention

After discharge, participants were randomly assigned to LGL dietary intervention group or control group. Each received specially prepared Diabetes Diet manual from the Clinical Nutrition Department, with an energy target generally around 25 kcal/kg of ideal body weight (for most of them were overweight and engaged in light physical activities) and a macronutrient distribution of 45%–55% carbohydrate, 15%–20% protein, and 25%–35% fat. This moderate energy restriction and distribution was designed to improve glycemic control, reduce adiposity, achieve gradual and sustainable metabolic improvement and help for long‐term adherence to diet, without excessive calorie deprivation and rapid weight loss. Control group received manual listing typical Chinese diet options with medium to high GI foods, while the intervention group received manual focusing on low GI foods to achieve a LGL regimen. GI values were obtained from the internationally published GI table and the *Chinese Food Composition Standard Edition* (6th Edition 2018) (Yang 2018). Intervention group used GL calculation software and recipes provided by nutritionists to select predominantly low‐GI foods, such as whole grains and beans accounting for half of the staple food, 500 g of nonroot vegetables, and 150 g of low‐GI fruits per day, which resulted in a low overall dietary GI (< 55). Based on these actual low‐GI food compositions and the final energy range of 1100–1800 kcal/day (related to the fact that the body types of the Chinese population are generally smaller compared to those of the Caucasian populations), participants were required to manage daily dietary GL between 75 and 125 and GL/1000 kcal between 56 and 68. GI and GL‐related knowledge were reinforced through questionnaires and educational sessions conducted every 3 months during follow‐up visits. Compliance was monitored by requiring all participants to upload their daily food intake on the social media platform “Wechat” at least 2 days a week, including 1 weekday and 1 weekend day, and to submit a 3‐day dietary record form (including 2 weekdays and 1 weekend day) during their monthly hospital visits for the first 3 months, followed by quarterly visits thereafter until the phase of 1‐year follow‐ups.

Key metabolic and biometric assessments, including HbA1c and lipid profiles, were conducted every 3 months. Participants also monitored their fingertip blood glucose at home every 3 days a week and four times a day (including fasting and 2 h postmeals glucose) or used dynamic blood glucose monitoring for 2 weeks per month, avoided any glucose or lipid altering drugs before study exit, and maintained their usual exercise patterns throughout the study.

### 2.6 Data Collection

#### 2.6.1 General Investigation

A uniformly designed questionnaire was utilized to gather essential information from the participants. Relevant human parameters, including height (Ht, cm), weight (Wt, kg), waist circumference (Wc, cm), systolic blood pressure (SBP, mmHg), diastolic blood pressure (DBP, mmHg), and exercise data, were measured. These measurements were critical for assessing the participants' physical health status and were integral to the overall data collection process in the study.

#### 2.6.2 Laboratory Tests

Plasma glucose (PG) levels were determined using the hexokinase method. Total cholesterol (TC) and TG were measured by the enzyme reagent method and the phosphoglycerol oxidase method, respectively. HDL‐C and LDL‐C concentrations were determined using the homogeneous method. Liver and kidney functions were evaluated by measuring alanine aminotransferase (ALT) with the reductive coenzyme method, total bile acid (TBA) with the enzymatic cycling method, and serum creatinine (Scr) with the sarcosine oxidase method. Thyroid‐stimulating hormone (TSH) and insulin levels were determined by chemiluminescent immunoassay. All these tests were performed on a Beckman Coulter AU Biochemical Analyzer (AU5800, Beckman Coulter Corporation, USA). Additionally, glycosylated hemoglobin (HbA1c) concentrations were measured via high‐pressure liquid chromatography using the Variant II HbA1C High Protein Detector (BOLE, USA). Glycated albumin (GA) levels were determined using the enzyme reagent method on an automatic biochemical instrument BS‐600 (Mindray, China). The biochemical indexes maintained a coefficient of variation within and between batches of 2% and 3%, respectively.

#### 2.6.3 Calculated Indexes

1. *BMI*: This is calculated using the formula weight (kg)/height^2^ (m^2^).
2. WWI = waist circumference (cm)/square root of body weight (kg).
3. *Time in range (TIR) of blood glucose*: This represents the percentage of time that blood glucose levels remain within the target range of 3.9–10.0 mmol/L over a 24‐h period.
4. *VAI*: for male: $\mathrm{VAI}=\frac{\mathrm{Wc}}{39.68+\left(1.88\times \mathrm{BMI}\right)}\times \frac{\mathrm{TG}}{1.03}\times \frac{1.31}{\mathrm{HDL}-C}$; for female: $\mathrm{VAI}=\frac{\mathrm{Wc}}{36.58+\left(1.89\times \mathrm{BMI}\right)}\times \frac{\mathrm{TG}}{0.81}\times \frac{1.52}{\mathrm{HDL}-C}$.
5. AI = (TC – HDL − C)/HDL − C.
6. *Dietary indexes*: GL = Σ (GI of food × carbohydrate content of food consumed)/100; Energy‐adjusted GL (GL/1000 kcal) = GL/(Average daily energy intake/1000 kcal).
7. *Metabolic equivalent hours per week (MET‐h/week)*: This metric is calculated based on the frequency and duration of the activity. Specifically, the time spent performing the activity is multiplied by the MET value corresponding to that activity. Physical activity levels are classified as follows: less than 10 MET‐h/week is considered low physical activity; 10–50 MET‐h/week is classified as moderate exercise; more than 50 MET‐h/week is regarded as high physical activity.

### 2.7 Quality Control

All investigators received standardized training to ensure adherence to the research protocol and standard operating procedures (SOPs). To minimize recall bias, participants were asked to record their 3‐day dietary intake on the day of consumption itself. Follow‐up assessments were scheduled within a predefined period of 7–14 days. Importantly, to prevent any cross‐contamination of results, follow‐up sessions for the intervention group and the control group were conducted separately. Blood samples were collected and handled strictly according to the hospital's Specimen Collection Manual to maintain consistency and integrity. After data collection, all survey responses were entered and verified using unified software (EpiData) through a process of online double input. Additionally, personnel from the clinical trial center conducted regular reviews of the database to verify and ensure the accuracy of the data.

### 2.8 Statistical Analysis

All statistical analyses were performed using R language (version 4.3.2). For categorical data, the number of cases (%) were used for descriptions, and comparisons were made using the *χ* ^2^ test or Fisher's exact probability method when appropriate. For quantitative data, those conforming to a normal distribution were expressed as mean (standard deviation) and compared using the *t*‐test. Quantitative data not following a normal distribution were described as median (P25, P75) and analyzed using the rank sum test for intergroup comparisons. For repeated measurement data, such as biochemical indicators, the study faced issues with missing data during follow‐up; thus, the generalized estimation equation model from the geepack package in R was used to estimate differences between groups. *p* < 0.05 was considered statistically significant in all analyses.

## 3 Results

**FIGURE 2:**
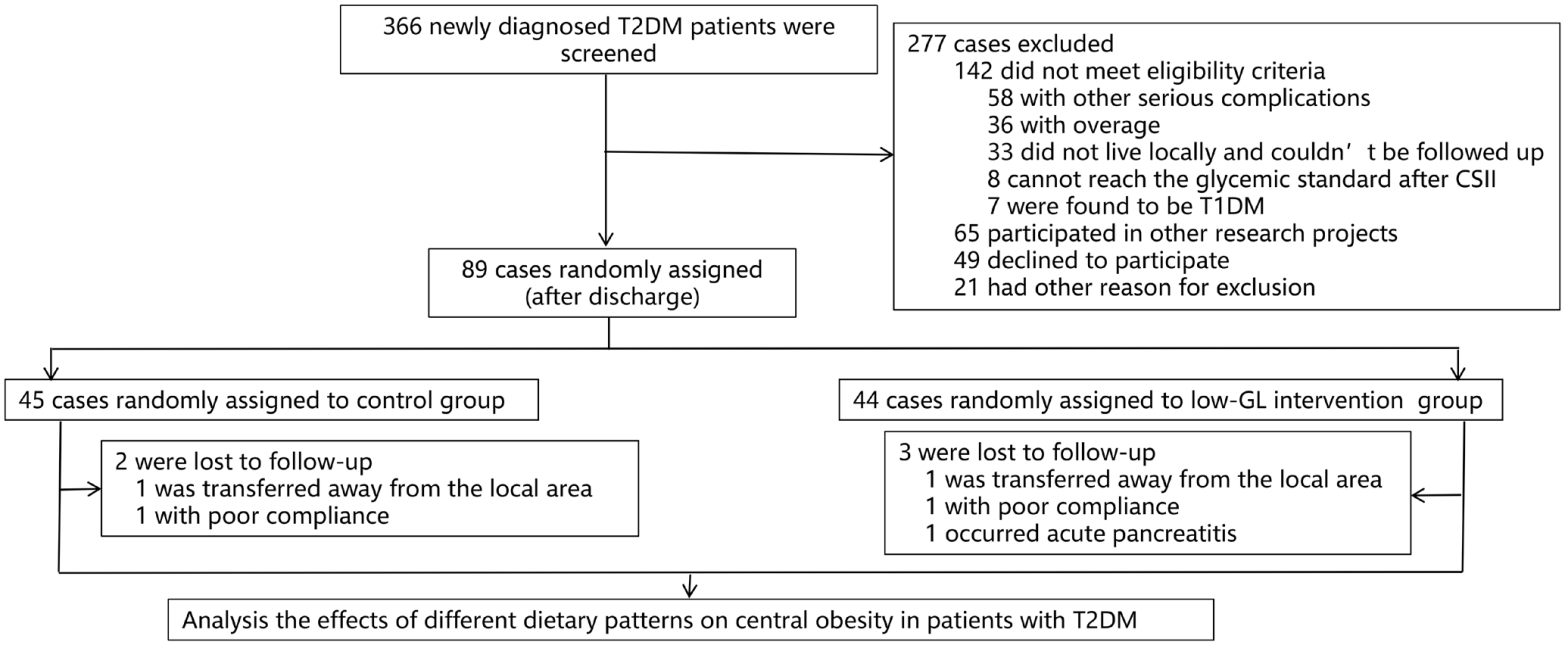
Trial profile. A total of 366 patients with newly diagnosed T2DM were screened. Finally, 89 patients were successfully enrolled and included in the FAS, of which 45 patients were randomly selected into the control group and 44 patients into the low‐GL diet intervention group. During the follow‐up period, two patients from the control group and three from the low‐GL group withdrew from the study. CSII: continuous subcutaneous insulin infusion, GL: glycemic load, T1DM: Type 1 diabetes mellitus, T2DM: Type 2 diabetes mellitus.

Between January 2019 and December 2023, 366 newly diagnosed patients with T2DM were screened. From this cohort, 45 patients were randomly assigned to the control group and 44 to the LGL dietary intervention group, resulting in a total of 89 patients included in the analytic set. The flowchart detailing the inclusion and screening process for research participants is provided in Figure 2.

### 3.1 Changes of Clinical Indicators Before and After SIIT

As demonstrated in Table 1, significant improvements were observed in various clinical indicators of all enrolled patients after treatment with SIIT. These improvements included HbAlc, GA, Wt, BMI, Wc, blood pressure (SBP/DBP), and lipid profiles (TC, TG, HDL‐C, LDL‐C) (*p* < 0.05). Although most parameters were generally ameliorated after SIIT, many patients still exhibited mild residual metabolic abnormalities, such as slightly elevated blood glucose, suboptimal lipid levels, and persistent central obesity. Some interindividual variability was also observed in the magnitude of improvement.

**TABLE 1:** Changes of clinical indicators before and after CSII in enrolled patients.

Variables (*n* = 89)	−2 weeks	0 week	*p*
HbAlc (%)	**10.90 (9.40, 12.10)**	**9.28 (1.52)**	< 0.001
GA (%)	**28.44 (8.43)**	**20.00 (16.75, 23.00)**	< 0.001
Wt (kg)	72.38 (14.35)	71.81 (13.83)	< 0.001
BMI (kg/m^2^)	**25.26 (23.51, 28.40)**	**24.85 (23.59, 27.85)**	< 0.001
Wc (cm)	**92.00 (87.00, 98.00)**	**90.25 (86.50, 97.62)**	< 0.001
WWI	**10.91 (0.62)**	**10.69 (10.22, 11.15)**	0.026
SBP (mmHg)	128.09 (15.19)	120.02 (11.92)	< 0.001
DBP (mmHg)	79.81 (11.80)	75.21 (8.13)	< 0.001
TC (mmol/L)	5.60 (1.14)	4.66 (1.01)	< 0.001
TG (mmol/L)	1.60 (1.25, 2.38)	1.47 (1.10, 1.91)	0.003
HDL‐C (mmol/L)	**1.02 (0.91, 1.16)**	1.11 (0.97, 1.25)	< 0.001
LDL‐C (mmol/L)	**3.67 (0.88)**	**3.00 (0.78)**	< 0.001
VAI	**2.20 (1.57, 3.61)**	**2.07 (1.31, 2.54)**	0.002
AI	**4.20 (3.18, 4.90)**	**3.48 (1.08)**	< 0.001

*Note:* Bold values indicate results outside the normal reference range. Values are represented as mean (SD) or median (IQR). Chinese clinical laboratory standards and population‐specific criteria: HbA1c: 4%–6%; GA: 11%–16%; BMI: 18.5–23.9 kg/m^2^; Wc: male < 85 cm, female < 80 cm; SBP: < 140 mmHg; DBP: < 90 mmHg; TC: 2.9–6.0 mmol/L; TG: 0.56–2.3 mmol/L; HDL‐C: 1.03–2.07 mmol/L; LDL‐C: < 2.6 mmol/L. International criteria: WWI: male < 10.4, female < 10.5; VAI: male < 2.0, female < 1.5; AI: < 3.0.

Abbreviations: AI: atherosclerosis index, BMI: body mass index, CSII: continuous subcutaneous insulin infusion, DBP: diastolic blood pressure, GA: glycated albumin, HbA1c: glycated hemoglobin A1c, HDL‐C: high density lipoprotein cholesterol, LDL‐C: low density lipoprotein cholesterol, SBP: systolic blood pressure, TC: total cholesterol, TG: triglyceride, VAI: visceral adiposity index, Wc: waist circumference, WWI: Weight‐adjusted waist index.

### 3.2 Comparison of Baseline Data Between Two Groups Before Randomization

From the information provided in Table 2, it is evident there were no significant differences in demographic characteristics or clinical data after SIIT treatment between the two groups (*p* > 0.05). This suggests a consistent baseline across both groups prior to the commencement of the interventions, providing a reliable foundation for comparing the subsequent effects of the two dietary interventions.

**TABLE 2:** Comparison of baseline indicators between the two groups of patients.

Variables	Total (*n* = 89)	Control (*n* = 45)	Intervention (*n* = 44)	*p*
Age (y)	45.87 (10.71)	46.71 (9.89)	45.00 (11.53)	0.455
Sex, *n* (%)				0.572
Male	69 (77.5)	36 (80)	33 (75)	
Female	20 (22.5)	9 (20)	11 (25)	
Education, *n* (%)				0.347
Junior high and below	30 (33.7)	18 (40)	12 (27.3)	
Senior high to junior college	32 (36)	16 (35.6)	16 (36.4)	
University or above	27 (30.3)	11 (24.4)	16 (36.4)	
Occupation, *n* (%)				0.479
Worker	43 (48.3)	18 (40.0)	25 (56.8)	
Self‐employ	33 (37.1)	20 (44.4)	13 (29.6)	
Retirement	13 (14.6)	7 (15.6)	6 (13.6)	
Health care system, *n* (%)				0.808
Urban medical insurance	72 (80.9)	38 (84.4)	34 (77.3)	
Rural medical service	17 (19.1)	7 (15.6)	10 (22.7)	
Duration of diabetic symptoms (m)	2.00 (0.50, 8.00)	2.00 (1.00, 16.00)	2.00 (0.19, 7.25)	0.451
Duration of diabetic diagnosis (m)	0.30 (0.07, 1.50)	0.25 (0.07, 1.25)	0.38 (0.10, 4.00)	0.364
History of hypertension, *n* (%)				0.883
No	69 (78.4)	35 (77.8)	34 (79.1)	
Yes	19 (21.6)	10 (22.2)	9 (20.9)	
History of dyslipidemia, *n* (%)				0.082
No	76 (85.4)	40 (88.9)	36 (81.8)	
Yes	13 (14.6)	5 (11.1)	8 (18.2)	
History of thyropathy, *n* (%)				1.000
No	81 (91)	41 (91.1)	40 (90.9)	
Yes	8 (9)	4 (8.9)	4 (9.1)	
History of smoking, *n* (%)				0.227
No	53 (59.6)	24 (53.3)	29 (65.9)	
Yes	36 (40.4)	21 (46.7)	15 (34.1)	
Menstruation, *n* (%)				0.257
Male	69 (77.5)	36 (80)	33 (75)	
Menopause	14 (15.7)	8 (17.8)	6 (13.6)	
Premenopause	6 (6.7)	1 (2.2)	5 (11.4)	
Family history of diabetes, *n* (%)				0.457
No	44 (49.4)	24 (53.3)	20 (45.5)	
Yes	45 (50.6)	21 (46.7)	24 (54.5)	
Family history of hypertension, *n* (%)				0.763
No	64 (71.9)	33 (73.3)	31 (70.5)	
Yes	25 (28.1)	12 (26.7)	13 (29.5)	
Dietary energy intake (kcal)	2342.14 (669.09)	2301.78 (638.66)	2383.42 (703.83)	0.568
Dietary CHO intake (g)	303.30 (100.54)	294.13 (102.73)	312.67 (98.53)	0.387
Dietary GL	215.83 (80.41)	213.97 (79.67)	217.73 (82.04)	0.827
Dietary GL/1000 kcal	91.55 (19.96)	92.02 (16.57)	91.07 (23.11)	0.824
Dietary CHO%	54.49 (8.08)	53.58 (8.67)	55.42 (7.40)	0.285
Dietary PRO%	16.33 (3.74)	16.83 (4.36)	15.81 (2.94)	0.200
Dietary FAT%	29.37 (6.61)	29.68 (6.46)	29.06 (6.83)	0.662
MET‐h/week	10.29 (3.73)	10.50 (3.79)	10.06 (3.71)	0.581
Duration of CSII (days)	12 (11, 14)	13 (11, 15)	12 (11, 13)	0.211
Mean insulin of base rate (U)	18.20 (14.70, 22.75)	18.50 (15.30, 22.70)	17.80 (14.53, 23.08)	0.563
Mean insulin of bolus (U)	22.44 (7.65)	22.78 (8.05)	22.11 (7.33)	0.692
Reach glycemic standard (days)	5.00 (2.00, 6.00)	5.00 (2.75, 6.00)	4.50 (2.00, 6.00)	0.791
Duration of euglycemia (days)	8.00 (6.00, 10.00)	8.00 (6.00, 10.00)	7.50 (6.00, 9.25)	0.930
TIR (3.9–10 mmol/L, %)	75.86 (68.66, 85.94)	75.90 (69.01, 85.92)	75.43 (68.65, 86.04)	0.964
Hypoglycemic frequency (*n*)	3 (1, 4)	2 (1, 3)	3 (1, 5)	0.451
BMI (kg/m^2^)	**24.85 (23.59, 27.85)**	**25.18 (23.89, 27.75)**	**24.59 (23.23, 27.86)**	0.455
Wc (cm)	**90.25 (86.50, 97.62)**	**93.00 (85.62, 98.75)**	**89.00 (86.50, 94.25)**	0.429
WWI	**10.69 (10.22, 11.15)**	**10.77 (10.24, 11.14)**	**10.64 (10.20, 11.12)**	0.508
SBP (mmHg)	120.02 (11.92)	122.23 (12.27)	117.77 (11.25)	0.081
DBP (mmHg)	75.21 (8.13)	77.02 (7.80)	73.35 (8.13)	0.054
ALT (U/L)	27.00 (20.00, 40.00)	30.50 (23.00, 41.75)	23.00 (17.00, 33.50)	0.106
TBA (μmol/L)	3.60 (2.50, 7.12)	4.60 (2.40, 7.10)	3.50 (2.95, 6.55)	0.830
Scr (μmol/L)	81.93 (13.61)	81.85 (13.06)	82.00 (14.28)	0.961
TSH (mU/L)	1.31 (0.98, 2.13)	1.38 (1.04, 2.06)	1.18 (0.96, 2.16)	0.540
HbAlc (%)	**9.28 (1.52)**	**9.37 (1.53)**	**9.19 (1.54)**	0.593
GA (%)	**20.00 (16.75, 23.00)**	**20.00 (16.00, 24.00)**	**21.00 (18.00, 23.00)**	0.977
TC (mmol/L)	4.66 (1.01)	4.47 (1.00)	4.84 (0.99)	0.093
TG (mmol/L)	1.47 (1.10, 1.91)	1.44 (1.04, 1.66)	1.64 (1.13, 2.25)	0.102
HDL‐C (mmol/L)	**1.02 (0.91, 1.16)**	**0.99 (0.90, 1.16)**	**1.04 (0.94, 1.14)**	0.414
LDL‐C (mmol/L)	**3.00 (0.78)**	**2.86 (0.79)**	**3.14 (0.76)**	0.110
VAI	**2.07 (1.31, 2.54)**	**1.85 (1.39, 2.39)**	**2.34 (1.02, 2.74)**	0.609
AI	**3.48 (1.08)**	**3.35 (1.14)**	**3.61 (1.02)**	0.270

*Note:* Data were expressed as means (standard deviation) or median (interquartile range). Bold values indicate results outside the normal reference range. Chinese clinical laboratory standards and population‐specific criteria: BMI: 18.5–23.9 kg/m^2^; Wc: male < 85 cm, female < 80 cm; SBP: < 140 mmHg; DBP: < 90 mmHg; ALT: 7–40 U/L; TBA: 0‐10 μmol/L; Scr: 57–97 μmol/L; TSH: 0.55–4.78 mU/L; HbA1c: 4%–6%; GA: 11%–16%; TC: 2.9–6.0 mmol/L; TG: 0.56–2.3 mmol/L; HDL‐C: 1.03–2.07 mmol/L; LDL‐C: < 2.6 mmol/L. International criteria: WWI: male < 10.4, female < 10.5; VAI: male < 2.0, female < 1.5; AI: < 3.0.

Abbreviations: AI: atherosclerosis index, ALT: alanine aminotransferase, BMI: body mass index, CHO%: percentage of energy provided by carbohydrates, CHO: carbohydrate, CSII: continuous subcutaneous insulin infusion, DBP: diastolic blood pressure, FAT%: percentage of energy provided by fat, GA: glycated albumin, GL/1000 kcal: energy‐corrected GL, GL: glycemic load, HbA1c: glycated hemoglobin, HDL‐C: high‐density lipoprotein cholesterol, LDL‐C: low‐density lipoprotein cholesterol, MET‐h/week: metabolic equivalent number of hours per week, PRO%: percentage of energy provided by protein, SBP: systolic blood pressure, Scr: serum creatinine, TBA: total bile acid, TC: total cholesterol, TG: triglycerides, TIR: time in range (3.9–10 mmol/L), TSH: thyroid stimulating hormone, VAI: visceral adiposity index, Wc: waist circumference, WWI: weight‐adjusted waist circumference index.

### 3.3 Dietary Intervention During Follow‐Up After Randomization in Two Groups

Throughout the follow‐up period postdischarge, significant reductions were observed in actual dietary energy intake, GL/1000 kcal and CHO% in both the control group and the LGL group compared to baseline assessed by dietary surveys (*p* < 0.05, *p*_time_ < 0.001). Conversely, the PRO% and FAT% were significantly higher than baseline levels in both groups (*p* < 0.05, *p*_time_ < 0.001). Statistically significant differences were found between the two groups in dietary GL/1000 kcal, CHO%, PRO% and FAT% over time (*p*_group_ < 0.05, *p*_group_ × time < 0.05), despite the similarity in energy intake between the groups (*p*_group_ > 0.05). These changes were noted in Table 3.

**TABLE 3:** Clinical characteristics of patients in both groups at various time points after randomization.

Time (m)	Control	Low‐GL	Mean difference of control (95% CI)	Mean difference of low‐GL (95% CI)	Difference between groups	Group	Time	Group × time
*Energy (kcal)*						*χ* ^2^ = 3.515, *p* = 0.061	*χ* ^2^ = 135.985, *p* < 0.001***	*χ* ^2^ = 14.540, *p* = 0.024*
0	2300.08 (57.83)	2364.49 (63.51)	—	—	64.42 (−76.17, 205.00)			
1	1630.73 (99.15)	1504.69 (74.99)	−669.35 (−924.21, −414.48)***	−859.81 (−1045.52, −674.10)***	−190.46 (−504.98, 124.06)			
2	1554.63 (113.44)	1588.57 (70.59)	−745.45 (−1004.44, −486.45)***	−775.93 (−937.47, −614.38)***	−30.48 (−337.77, 276.81)			
3	1950.18 (81.52)	1658.59 (77.31)	−349.90 (−497.96, −201.85)***	−705.91 (−896.29, −515.53)***	−356.00 (−597.18, −114.83)**			
6	1756.17 (104.40)	1661.08 (80.27)	−543.91 (−791.63, −296.19)***	−703.41 (−919.96, −486.86)***	−159.50 (−486.84, 167.83)			
9	1765.54 (132.18)	1612.55 (81.98)	−534.53 (−825.04, −244.03)***	−751.95 (−936.93, −566.96)***	−217.41 (−562.82, 128.00)			
12	1819.39 (82.90)	1576.85 (60.12)	−480.68 (−680.12, −281.25)***	−787.65 (−956.72, −618.57)***	−306.96 (−571.71, −42.21)*			
*GL (1000 kcal)*						*χ* ^2^ = 57.463, *p* < 0.001***	*χ* ^2^ = 83.951, *p* < 0.001***	*χ* ^2^ = 51.694, *p* < 0.001***
0	94.81 (2.22)	89.93 (2.42)	—	—	−4.88 (−10.74, 0.99)			
1	77.02 (4.12)	59.99 (3.10)	−17.79 (−26.60, −8.98)***	−29.95 (−38.88, −21.01)***	−12.16 (−24.75, 0.43)*			
2	77.56 (3.78)	63.79 (3.07)	−17.25 (−25.74, −8.76)***	−26.14 (−34.65, −17.64)***	−8.89 (−21.03, 3.25)			
3	87.92 (2.15)	58.55 (1.67)	−6.88 (−12.12, −1.65)*	−31.38 (−38.36, −24.40)***	−24.50 (−33.22, −15.77)***			
6	70.82 (4.70)	60.77 (2.52)	−23.99 (−34.34, −13.64)***	−29.16 (−37.28, −21.04)***	−5.17 (−18.28, 7.95)			
9	82.62 (6.16)	68.09 (3.55)	−12.19 (−24.93, 0.56)	−21.85 (−31.42, −12.27)***	−9.66 (−25.47, 6.15)			
12	69.79 (4.66)	61.62 (4.47)	−25.02 (−34.22, −15.81)***	−28.31 (−39.13, −17.49)***	−3.29 (−17.41, 10.83)			
*CHO%*						*χ* ^2^ = 11.295, *p* = 0.001**	*χ* ^2^ = 64.820, *p* < 0.001***	*χ* ^2^ = 25.804, *p* < 0.001***
0	54.49 (0.98)	54.94 (0.88)	—	—	0.45 (−1.92, 2.82)			
1	53.24 (1.50)	48.91 (0.84)	−1.25 (−5.00, 2.50)	−6.03 (−8.81, −3.24)***	−4.77 (−9.43, −0.12)*			
2	51.69 (1.75)	49.36 (0.78)	−2.80 (−6.59, 0.99)	−5.58 (−7.97, −3.19)***	−2.78 (−7.26, 1.70)			
3	52.40 (0.98)	45.10 (0.97)	−2.08 (−4.10, −0.07)*	−9.83 (−12.80, −6.87)***	−7.75 (−11.33, −4.17)***			
6	46.59 (1.85)	45.22 (1.15)	−7.89 (−12.14, −3.65)***	−9.72 (−13.03, −6.41)***	−1.82 (−7.22, 3.58)			
9	50.80 (2.23)	47.81 (1.60)	−3.69 (−8.42, 1.04)	−7.13 (−11.26, −2.99)**	−3.44 (−9.65, 2.78)			
12	48.16 (1.57)	46.76 (1.41)	−6.33 (−9.85, −2.81)***	−8.18 (−12.01, −4.34)***	−1.85 (−6.97, 3.28)			
*PRO (%)*						*χ* ^2^ = 16.594, *p* < 0.001***	*χ* ^2^ = 80.032, *p* < 0.001***	*χ* ^2^ = 27.607, *p* < 0.001***
0	15.88 (0.38)	16.17 (0.34)	—	—	0.30 (−0.57, 1.17)			
1	18.07 (0.85)	20.26 (0.56)	2.19 (0.37, 4.02)*	4.08 (2.57, 5.59)***	1.89 (−0.48, 4.25)			
2	18.91 (1.12)	18.70 (0.50)	3.03 (0.86, 5.20)**	2.52 (1.19, 3.86)***	−0.51 (−3.05, 2.04)			
3	17.33 (0.51)	21.75 (0.58)	1.45 (0.35, 2.55)*	5.57 (4.07, 7.08)***	4.12 (2.26, 5.99)***			
6	18.95 (0.87)	20.42 (0.79)	3.07 (1.07, 5.07)**	4.25 (2.46, 6.04)***	1.18 (−1.49, 3.85)			
9	17.37 (1.00)	19.89 (0.81)	1.50 (−0.53, 3.52)	3.72 (2.05, 5.39)***	2.22 (−0.38, 4.83)			
12	20.61 (0.67)	21.55 (0.74)	4.73 (3.40, 6.06)***	5.38 (3.90, 6.86)***	0.65 (−1.28, 2.59)			
*FAT (%)*						*χ* ^2^ = 4.079, *p* = 0.043*	*χ* ^2^ = 26.370, *p* < 0.001***	*χ* ^2^ = 12.153, *p* = 0.059
0	29.64 (0.77)	29.26 (0.77)	—	—	−0.37 (−2.40, 1.65)			
1	30.71 (1.16)	31.52 (0.64)	1.08 (−2.08, 4.23)	2.25 (0.00, 4.50)	1.18 (−2.68, 5.03)			
2	31.52 (1.18)	33.42 (0.66)	1.88 (−1.11, 4.88)	4.15 (2.16, 6.15)***	2.27 (−1.33, 5.87)			
3	30.64 (0.79)	34.35 (0.77)	1.00 (−0.70, 2.70)	5.09 (2.55, 7.62)***	4.09 (1.04, 7.14)**			
6	33.06 (2.42)	34.68 (0.90)	3.42 (−1.40, 8.25)	6.42 (3.81, 9.03)**	2.99 (−2.56, 8.55)			
9	33.36 (1.64)	33.54 (1.43)	3.72 (−0.03, 7.47)	4.28 (0.56, 7.99)*	0.56 (−4.66, 5.77)			
12	32.76 (1.93)	32.31 (1.41)	3.12 (−1.20, 7.44)	3.05 (−0.73, 6.82)	−0.07 (−5.80, 5.65)			
*BMI* (*kg/m* ^*2*^)						*χ* ^2^ = 1.114, *p* = 0.291	*χ* ^2^ = 20.336, *p* = 0.002**	*χ* ^2^ = 9.356, *p* = 0.155
0	**25.64 (0.10)**	**25.69 (0.07)**	—	—	0.05 (−0.11, 0.21)			
1	**25.39 (0.18)**	**25.16 (0.11)**	−0.25 (−0.57, 0.08)	−0.53 (−0.76, −0.31)***	−0.29 (−0.68, 0.11)			
2	**25.70 (0.27)**	**24.99 (0.15)**	0.06 (−0.45, 0.58)	−0.70 (−1.01, −0.40)***	−0.77 (−1.37, −0.17)*			
3	**25.47 (0.17)**	**24.90 (0.17)**	−0.17 (−0.49, 0.15)	−0.79 (−1.12, −0.45)***	−0.62 (−1.08, −0.15)*			
6	**25.81 (0.44)**	**24.77 (0.23)**	0.17 (−0.69, 1.03)	−0.92 (−1.36, −0.48)***	−1.09 (−2.06, −0.12)*			
9	**25.58 (0.50)**	**24.59 (0.28)**	−0.06 (−1.08, 0.95)	−1.10 (−1.63, −0.57)***	−1.04 (−2.18, 0.11)			
12	**25.60 (0.35)**	**24.94 (0.32)**	−0.04 (−0.74, 0.66)	−0.75 (−1.37, −0.14)*	−0.71 (−1.65, 0.22)			
*Wc (cm)*						*χ* ^2^ = 0.449, *p* = 0.503	*χ* ^2^ = 47.309, *p* < 0.001***	*χ* ^2^ = 10.175, *p* = 0.117
0	**90.46 (0.51)**	**90.66 (0.38)**	—	—	0.20 (−0.48, 0.88)			
1	**87.00 (1.17)**	**87.73 (0.74)**	−3.46 (−5.55, −1.36)**	−2.92 (−4.19, −1.66)***	0.53 (−1.88, 2.94)			
2	**88.83 (1.63)**	**87.72 (0.83)**	−1.63 (−4.57, 1.31)	−2.94 (−4.40, −1.48)***	−1.31 (−4.59, 1.97)			
3	**88.25 (1.10)**	**86.13 (0.82)**	−2.21 (−4.08, −0.33)*	−4.53 (−5.90, −3.16)***	−2.32 (−4.64, −0.00)			
6	**89.32 (2.03)**	**85.86 (0.98)**	−1.14 (−4.88, 2.60)	−4.80 (−6.60, −3.00)***	−3.66 (−7.82, 0.50)			
9	**87.89 (1.59)**	**86.18 (1.08)**	−2.57 (−5.48, 0.33)	−4.48 (−6.52, −2.45)***	−1.91 (−5.47, 1.65)			
12	**87.90 (1.32)**	**86.71 (0.99)**	−2.56 (−5.07, −0.05)*	−3.95 (−5.84, −2.07)***	−1.40 (−4.54, 1.75)			
*WWI*						*χ* ^2^ = 0.281, *p* = 0.596	*χ* ^2^ = 13.461, *p* = 0.007**	*χ* ^2^ = 2.536, *p* = 0.864
0	10.08 (0.16)	10.48 (0.34)	—	—	0.41 (−1.53, 0.71)			
1	9.97 (0.18)	10.41 (0.34)	−0.08 (−0.11, 0.02)	−0.04 (−0.08, 0.02)	0.56 (−1.10, 2.23)			
2	9.89 (0.37)	9.92 (0.38)	−0.13 (−0.18, −0.05)*	−0.54 (−0.87, −0.24)*	0.04 (−1.03, 1.38)			
3	9.77 (0.17)	9.73 (0.21)	−0.16 (−0.21, −0.09)*	−0.76 (−0.91, −0.31)**	−0.03 (−0.72, 2.20)			
6	9.73 (0.22)	9.52 (0.21)	−0.16 (−0.22, −0.09)*	−0.95 (−1.34, −0.55)**	−0.20 (−1.03, 1.42)			
9	9.48 (0.44)	9.16 (0.20)	−0.58 (−0.72, −0.38)**	−1.34 (−1.87, −0.95)***	−0.36 (−0.91, 2.04)			
12	9.32 (0.30)	9.05 (0.19)	−0.72 (−0.99, −0.58)**	−1.45 (−1.86, −1.03)***	−0.24 (−0.72, 2.39)			
*HbAlc (%)*						*χ* ^2^ = 4.108, *p* = 0.043*	*χ* ^2^ = 250.744, *p* < 0.001***	*χ* ^2^ = 10.876, *p* = 0.028*
0	**9.49 (0.18)**	**9.44 (0.16)**	—	—	−0.05 (−0.52, 0.42)			
3	**6.58 (0.23)**	**6.09 (0.15)**	−2.91 (−3.60, −2.22)***	−3.35 (−3.85, −2.85)***	−0.44 (−1.28, 0.40)			
6	**6.17 (0.27)**	**6.26 (0.25)**	−3.33 (−4.06, −2.59)***	−3.18 (−3.86, −2.49)***	0.15 (−0.85, 1.14)			
9	**7.70 (0.43)**	**6.24 (0.23)**	−1.79 (−2.66, −0.93)***	−3.21 (−3.79, −2.62)***	−1.41 (−2.44, −0.39)**			
12	**6.39 (0.35)**	5.94 (0.15)	−3.10 (−3.99, −2.22)***	−3.51 (−3.96, −3.06)***	−0.40 (−1.39, 0.58)			
*VAI*						*χ* ^2^ = 0.115, *p* = 0.735	*χ* ^2^ = 12.771, *p* = 0.012*	*χ* ^2^ = 3.792, *p* = 0.435
0	1.95 (0.21)	**2.20 (0.11)**	—	—	0.25 (−0.15, 0.64)			
3	1.36 (0.43)	1.24 (0.47)	−0.58 (−1.47, 0.30)	−0.95 (−1.86, −0.05)*	−0.37 (−1.75, 1.01)			
6	**2.02 (0.21)**	**2.51 (1.85)**	0.07 (−0.39, 0.52)	1.32 (−2.36, 4.99)	1.25 (−2.20, 4.70)			
9	1.86 (0.55)	1.38 (0.42)	−0.09 (−1.21, 1.03)	−0.81 (−1.67, 0.04)*	−0.72 (−2.18, 0.74)			
12	1.29 (0.28)	1.17 (0.73)	−0.66 (−1.32, 0.00)	−0.03 (−1.53, 1.47)	0.63 (−0.78, 2.05)			
*AI*						*χ* ^2^ = 1.009, *p* = 0.315	*χ* ^2^ = 11.465, *p* = 0.022*	*χ* ^2^ = 6.821, *p* = 0.146
0	**3.62 (0.09)**	**3.50 (0.08)**	—	—	−0.12 (−0.30, 0.07)			
3	2.60 (0.17)	**3.17 (0.26)**	−1.02 (−1.42, −0.62)***	−0.34 (−0.84, 0.17)	0.68 (0.03, 1.33)			
6	2.08 (0.65)	**3.15 (0.20)**	−1.54 (−2.88, −0.21)*	−0.35 (−0.79, 0.10)	1.20 (−0.21, 2.60)			
9	2.69 (0.49)	2.76 (0.26)	−0.93 (−1.95, 0.08)	−0.74 (−1.31, −0.16)*	0.19 (−0.97, 1.35)			
12	1.80 (0.71)	2.02 (0.28)	−1.82 (−3.29, −0.35)*	−0.49 (−1.05, 0.08)	1.33 (−0.24, 2.90)			
*MET‐h/week*						*χ* ^2^ = 1.662, *p* = 0.197	*χ* ^2^ = 46.475, *p* < 0.001***	*χ* ^2^ = 10.207, *p* = 0.116
0	9.70 (1.20)	9.75 (1.00)	—	—	0.04 (−1.63, 1.72)			
1	14.50 (3.88)	19.57 (4.03)	4.79 (−2.44, 12.02)	9.82 (2.19, 17.45)*	5.03 (−5.49, 15.54)			
2	8.66 (2.56)	9.09 (3.07)	−1.04 (−6.35, 4.26)	−0.65 (−6.25, 4.94)	0.39 (−7.32, 8.10)			
3	29.17 (3.56)	21.71 (3.22)	19.47 (12.10, 26.83)***	11.96 (5.56, 18.37)***	−7.50 (−17.20, 2.19)			
6	17.64 (4.62)	19.33 (3.42)	7.94 (−0.99, 16.87) 1	9.59 (3.31, 15.86)**	1.65 (−9.08, 12.37)			
9	10.74 (2.47)	13.77 (3.52)	1.04 (−3.93, 6.00)	4.02 (−2.76, 10.80)	2.98 (−5.02, 10.99)			
12	28.72 (7.20)	15.55 (3.67)	19.01 (4.51, 33.52)**	5.81 (−0.39, 12.01)	−13.21 (−28.73, 2.32)			

*Note:* The mean (standard error) was estimated using a generalized linear model, adjusted for age, sex, education, TIR, and baseline levels of the corresponding indicators. Bold values indicate results outside the normal reference range. Chinese clinical laboratory standards and population‐specific criteria: BMI: 18.5–23.9 kg/m^2^; Wc: male < 85 cm, female < 80 cm; HbA1c: 4%–6%. International criteria: WWI: male < 10.4, female < 10.5; VAI: male < 2.0, female < 1.5; AI: < 3.0.

Abbreviations: AI: atherosclerosis index, BMI: body mass index, CHO%/PRO%/FAT%: carbohydrate/protein/fat percentage of energy supply, CHO: carbohydrates, GL/1000 kcal: energy‐adjusted glycemic load, GL: glycemic load, HbA1c: glycated hemoglobin A1c, MET‐h/week: metabolic equivalent hours per week, VAI: visceral adiposity index, Wc: waist circumference, WWI: weight‐adjusted waist index.

* *p* < 0.05.

** *p* < 0.01.

*** *p* < 0.001.

### 3.4 Changes in Central Obesity and Lipid Related Metabolic Abnormalities During Follow‐Up

During the follow‐up period, as presented in Table 3, BMI, Wc, WWI, VAI, and AI of the patients in both groups were significantly reduced, while MET‐h/week was significantly increased (*p* < 0.05, *p*_time_ < 0.001), but there was no statistically significant difference observed between the groups (*p*_group_ > 0.05). The reduction in HbA1c level was more significant in the LGL group (*p*_time_ < 0.001, *p*_group_ < 0.05).

## 4 Discussion

This study supported that both LGL diet and conventional energy‐restricted diet can improve central obesity (WWI) and lipid‐related metabolic abnormalities (VAI and AI) in newly diagnosed T2DM patients after SIIT, which was similar to multiple RCT evidence, indicating that LGL diet, when implemented in conjunction with energy restriction, also has broad potential in managing obesity and metabolic abnormalities. For instance, studies (Manta et al. 2023; Wijewardhana et al. 2023) indicate that LGL diet can reduce the risk of metabolic complications by lowering blood glucose fluctuations and improving insulin sensitivity, which is in line with the improvement of WWI and AI in this study (WWI represents changes in abdominal fat distribution, while AI reflects improvements in lipid metabolism). Similarly, a systematic review (Kalaitzopoulou et al. 2023) emphasizes that LGL diet significantly improves anthropometric and cardiometabolic parameters in overweight/obese children, highlighting the effectiveness of GL management in long‐term interventions. However, it is worth noting that the differences in dietary regimens in this study did not lead to significant intergroup differences, suggesting that the additional benefits of GL reduction may be offset by energy restriction itself. The DIETFITS trial (Soto‐Mota et al. 2023) also observed a similar phenomenon: when both healthy low‐carbohydrate and healthy low‐fat diets significantly reduced GL, there was no significant difference in weight loss effects, and the mediating factors remain unclear, possibly related to the overall improvement in diet quality rather than a single nutrient. Overall, the conclusion of this study provides a flexible option for LGL diet in T2DM management, especially when combined with SIIT and moderate energy restriction, effectively alleviating central obesity and lipid disorders.

Compared with existing literature, the nondifferential results of this study between groups are consistent with some recent findings, but also remind us of the complexity of dietary intervention. A meta‐analysis (Di et al. 2025) included 19 low GI (Glycemic Index) diet and 4 LGL diet studies, finding that although these interventions significantly improved obesity‐related parameters, the effects of different dietary patterns often did not differ significantly, similar to conventional energy restriction (especially when the control group was a balanced energy‐restricted diet). Another meta‐analysis (Zhao et al. 2024) further pointed out that continuous energy restriction diets are superior to single‐component adjustments in weight loss and optimizing lipid metabolism, which explains why the conventional diet group in this study (energy restriction of 25–30 kcal/kg/day) was also effective. However, the advantages of LGL diet may be more prominent in specific mechanisms, that is reducing blood glucose fluctuations and enhancing blood glucose control, which may indirectly promote the normalization of lipid metabolism (such as the improvement of VAI and AI) (Gerontiti et al. 2024). Additionally, Kweh et al. (2023) mentioned that LGL diet as a treatment strategy for Prader–Willi syndrome patients can indirectly affect the obesity process by regulating insulin levels, providing support for the role of LGL diet in metabolic intervention. However, another study (Apekey et al. 2022) indicated that low‐carbohydrate diets improve HbA1c and lipids in the short term, but no intergroup differences were observed in this study, possibly because the control group also adopted a lower proportion of carbohydrates (45%–60%), or the effect being dominated by energy restriction rather than isolated GL reduction. In summary, the nondifferential results of this study emphasize that energy restriction is the core for improving metabolism in T2DM management, while LGL as an auxiliary strategy needs to be implemented on an individual basis within the framework of energy restriction.

When considering the possible mechanisms, the improvement of VAI and AI in this study may be related to the combined effects of energy restriction and GL reduction. Previous studies have shown that energy restriction itself can promote fat breakdown and reduce inflammation (Jayedi et al. 2023; Park et al. 2025), which explains the significant reduction in parameters after the baseline in both groups. Meanwhile, a LGL diet may alleviate IR by stabilizing blood glucose, thereby regulating lipid metabolism. Studies (Manta et al. 2023; Chekima et al. 2023) have emphasized that a high GL diet worsens IR and the risk of metabolic syndrome, while a LGL pattern improves insulin sensitivity and reduces liver lipid deposition (which explains the mechanism of AI improvement). Further studies (Qiu et al. 2024; Hullar et al. 2025) suggested that dietary patterns (such as the LGL regimen in this study) may alleviate dyslipidemia by modulating the gut microbiota or key enzymes (such as fatty acid synthesizing enzymes), consistent with the reduction in AI and VAI. This is also the direction we will further explore in the future.

### 4.1 Contributions to Existing Knowledge

The findings of this study contribute to the existing body of knowledge on nutritional intervention for newly diagnosed T2DM after SIIT by providing novel insights and practical advancements beyond previous work. First, unlike most previous studies that focused on the isolated effect of LGL diets or energy restriction alone, this study explores the effectiveness of LGL diet combined with moderate energy restriction in a specific population—newly diagnosed T2DM patients who have just completed SIIT. This study addresses a relatively underexplored area in post‐SIIT nutritional maintenance strategies, as few studies have specifically targeted this critical transition period when patients shift from in‐hospital intensive therapy to long‐term outpatient management, and our findings provide the first empirical framework for guiding clinical practice in this context. Second, this study confirms that both LGL and conventional energy‐restricted diets have comparable efficacy in improving central obesity and lipid metabolism, emphasizing that energy restriction is the core of metabolic improvement, while LGL can serve as a flexible auxiliary strategy. This finding complements existing meta‐analyses (Di et al. 2025; Zhao et al. 2024) by providing real‐world evidence for the clinical application of personalized nutritional interventions, especially for Chinese T2DM patients with distinct dietary patterns. Finally, the study clarifies the relationship between LGL intervention and energy restriction, avoiding the over‐interpretation of GL's independent effect, which helps to refine the understanding of dietary intervention mechanisms in T2DM management.

### 4.2 Limitations and Future Research Recommendations

The research has several limitations. First, although our hospital is one of the top three hospitals in endocrinology in South China, a single‐center study may still hinder the generalization of our findings. Second, when exploring the mechanism by which a LGL diet promotes the remission of DM, it was mainly analyzed through clinical‐related data, lacking in‐depth basic research at the animal or cell level.

Accordingly, several future directions are proposed to extend the current findings. First, multicenter studies with larger sample sizes can be conducted to improve the generalizability of the results to broader populations with newly diagnosed T2DM. Second, further basic research at the animal or cellular level is warranted to explore the underlying mechanisms linking LGL diet, energy restriction, and improvements in central obesity and lipid metabolism, such as insulin signaling, inflammatory pathways, or gut microbiota regulation. Third, longer follow‐up periods would be valuable to evaluate its impact on cardiovascular complication risk and long‐term metabolic stability. Fourth, future studies may explore individualized responses to LGL intervention to identify which subgroups of patients could derive greater benefit from this dietary pattern (e.g., patients with severe central obesity or poor glycemic control after SIIT). Finally, the development of simplified, clinically applicable LGL dietary tools (e.g., simplified GL calculation apps, personalized recipe databases) may help improve adherence and translate the current findings into routine clinical practice.

## 5 Conclusion

The results of this study emphasize that any effective energy‐restricted diet (whether LGL or conventional) can be used as a maintenance strategy after SIIT to manage central obesity and lipid abnormalities in newly diagnosed T2DM. These findings support the clinical benefit of an LGL diet when implemented in conjunction with energy restriction, rather than as an isolated GL‐lowering intervention, and provide foundational evidence for personalized nutritional intervention.

## Author Contributions

**Xiuhong Lin:** conceptualization, data curation, investigation, writing – original draft, formal analysis. **Xiaoting Lu:** software, validation, formal analysis. **Chaogang Chen:** methodology, funding acquisition, supervision, validation. **Diaozhu Lin:** methodology, investigation, resources. **Jin Zhang:** investigation, resources. **Chulin Huang:** methodology, investigation, resources. **Meng Ren:** methodology, investigation, visualization, resources. **Cheng Wang:** software, validation, formal analysis. **Xiaoyi Wang:** investigation, resources. **Mingtong Xu:** conceptualization, project administration, writing – review and editing, resources. **Dan Liu:** investigation, resources. **Li Yan:** conceptualization, project administration, writing – review and editing, resources.

## Funding

This research received funding from the National Natural Science Foundation of China (82373552), the Guangdong Clinical Research Center for Metabolic Diseases (2020B1111170009), the Guangzhou Key Laboratory for Metabolic Diseases (202102100004), the Sun Yat‐sen Clinical Research and Cultivation Project (SYS‐Q‐201904), and the Diabetes Mellitus Research Fund Program (DMRFP_II_08) provided by the Shanghai Medical and Health Development Foundation (SHMHDF).

## Ethics Statement

This project adhered to the Helsinki Declaration (seventh revision, 2013) and received approval from the Medical Ethics Committee of Sun Yat‐sen Memorial Hospital of Sun Yat‐sen University (No. 2018‐KY‐053).

## Consent

Participants were given details about the study and they provided signed informed consent.

## Conflicts of Interest

The authors declare no conflicts of interest.

## Data Availability

The data that support the findings of this study are available from the corresponding author upon reasonable request.
